# Potential Role of Oral Nitrate Bioactivation in Interindividual Exercise Responses to Dietary Nitrate: A Narrative Review

**DOI:** 10.3390/nu18203315

**Published:** 2026-10-09

**Authors:** Zhengxin Zhao, Zheng Lai, Shengwei Zhao, Wenli Li, Min Wang, Feng Guan, Xu Liang, Jiawei Zhang, Linding Wang

**Affiliations:** 1 Department of Sports, Nanjing University of Finance and Economics HongShan College, Nanjing 211300, China; 9120161014@nufe.edu.cn (Z.Z.); 8220210036@nufe.edu.cn (M.W.); 2 Laboratory of Gastrointestinal Microbiology, College of Animal Science and Technology, Nanjing Agricultural University, Nanjing 210095, China; zhenglai@stu.njau.edu.cn (Z.L.); 15850792155@163.com (S.Z.); guanfeng@stu.njau.edu.cn (F.G.); L3951570679@163.com (X.L.); zhang18043055977@163.com (J.Z.); 3 Anhui Provincial Key Laboratory of Zoonoses, Department of Microbiology and Parasitology, School of Basic Medical Sciences, Anhui Medical University, Hefei 230032, China; 2022510051@ahmu.edu.cn

**Keywords:** dietary nitrate, oral nitrate bioactivation, oral microbiome, nitrate-reducing bacteria, nitric oxide, salivary nitrite, exercise performance, interindividual variability, sports nutrition

## Abstract

**Background:** Equivalent doses of dietary nitrate often yield highly heterogeneous exercise responses across individuals. Efficient enterosalivary nitrate-to-nitrite conversion is dependent on oral commensal bacteria, representing a critical upstream step that may drive downstream variability in biomarker profiles and exercise outcomes. This review evaluates whether variation at the oral bioactivation step helps account for differences in downstream biomarkers and exercise performance. **Methods:** We conducted a structured narrative review of PubMed and Web of Science covering 1 January 2006 to 31 March 2026 using predefined keywords related to dietary nitrate, oral bioactivation, oral microbiota, enterosalivary pathway, and exercise performance. Human supplementation trials, antibacterial mouthwash perturbation studies, investigations evaluating oral function, and supporting case-report and preclinical evidence were included when they linked nitrate exposure, oral microbial activity or salivary nitrite kinetics, and physiological or exercise performance outcomes. **Results:** Oral bacteria are essential contributors to efficient enterosalivary nitrate-to-nitrite conversion. Associations between oral function and nitrite availability, as well as selected physiological measures, are reasonably consistent. However, direct evidence linking oral function to athlete performance remains sparse. No athlete trial identified in the present search has concurrently measured oral function, tracked serial nitrate/nitrite kinetics, and paired these data with a reliable performance test and prespecified mediation analysis. A rise in salivary nitrite therefore confirms engagement of the enterosalivary pathway, but it does not reliably identify an ergogenic responder. Current evidence does not yet allow quantitative estimation of how much between-person performance variability is attributable to oral bioactivation. **Conclusions:** Oral nitrate-reducing function remains a biologically plausible modifier of exercise responses, but it is not a validated predictive biomarker. Integrated athlete trials are needed to trace the full pathway from nitrate exposure to performance outcomes and to quantify within-person and between-person variation.

## 1. Introduction

Dietary nitrate, most commonly administered via beetroot juice or nitrate-rich vegetables, is a widely adopted ergogenic strategy in sports nutrition. However, findings from supplementation trials remain highly inconsistent [1,2,3,4]. Proposed mechanisms underlying nitrate’s ergogenic effects include improved vascular regulation and skeletal muscle oxygenation, enhanced mitochondrial efficiency, and modulated contractile function [5,6,7,8,9]. The magnitude of effects varies substantially across exercise modalities: meta-analytic evidence supports benefits for endurance and high-intensity intermittent protocols [1,4,10], whereas findings for single-sprint and resistance-based tasks are less consistent and may depend on dose, task format, and training status [11,12,13,14]. Multiple factors contribute to this heterogeneity, including dose and timing of supplementation, habitual dietary intake, and training status [3,4]. Biological sex and test reliability add further variation [15,16]. The practical research question is no longer merely whether nitrate can improve performance, but why comparable protocols produce clear effects in some people and negligible changes in others [1,2,3,4,5,6,11,12,17,18,19,20,21,22,23,24,25,26,27,28,29].

One underexplored source of this heterogeneity may reside upstream of systemic circulation and skeletal muscle. Humans have limited endogenous capacity to reduce nitrate to nitrite at physiologically meaningful rates. Following ingestion, nitrate is rapidly absorbed in the upper gastrointestinal tract; a substantial fraction of circulating nitrate is then actively taken up by the salivary glands and secreted into the oral cavity, where commensal bacteria reduce it to nitrite [7,30]. The tongue dorsum is a primary niche for this reductive activity, although saliva and dental plaque harbor distinct microbial communities that also contribute. Swallowed nitrite enters the systemic circulation and can be further reduced to bioactive nitric oxide, particularly under the hypoxic and acidic conditions encountered during intense exercise. Thus, nitrate dose only quantifies total exposure, while oral nitrate-reducing capacity is one major determinant of how much of that exposure is converted to bioactive product; salivary gland uptake, swallowing, gastric chemistry, systemic clearance, and background diet also contribute [7,8,9,30,31,32,33,34,35,36,37,38].

Two distinct bodies of review literature frame this problem. Sports nutrition reviews have comprehensively examined nitrate effects across exercise outcomes, while microbiome-focused papers have described the oral nitrate–nitrite–nitric oxide pathway and its broader physiological relevance. One recent narrative review explored links between gut microbiota and exercise-induced fatigue via metabolic, immune, and gut–brain pathways [39,40,41,42,43,44,45,46]. The overlap between these fields remains limited. The scope of the present review is specifically focused on whether the oral bioactivation step helps explain interindividual differences in exercise responses to dietary nitrate. This work is not intended as an exhaustive sport-by-sport catalogue of nitrate effects, nor a general review of the gut microbiome.

The full evidence chain linking oral function to exercise performance remains incomplete. While oral nitrate-reducing activity has been consistently associated with systemic nitrite availability, and supplementation trials report changes in select exercise outcomes, few studies have measured both endpoints in the same cohort. Oral microbial function is rarely assessed in parallel with serial nitrate/nitrite sampling and a validated performance test. As a result, a null performance finding cannot be unambiguously attributed to impaired oral bioactivation; it may equally reflect poor downstream translation of nitrite or an insensitive performance endpoint [26,36,38,40].

This gap can be framed as two distinct questions. First, does oral nitrate-reducing function influence nitrate/nitrite bioavailability? Second, does variation in that function explain variability in exercise performance after nitrate supplementation? The available evidence addresses the first question reasonably well, whereas direct evidence for the second remains very limited. Accordingly, this review pursues three objectives: (1) to establish what is known about oral nitrate bioactivation and how it should be measured; (2) to examine the human evidence connecting the oral step to exercise responses; and (3) to address individual response interpretation and propose a framework for future athlete trials. Throughout the review, biochemical, physiological, and performance responses are kept analytically separate, as evidence at one level does not constitute proof of an effect at the next. We hypothesize that interindividual differences in oral nitrate-reducing function contribute to variability in nitrate/nitrite bioavailability after nitrate ingestion, and that part of the heterogeneity in exercise performance responses to dietary nitrate may be attributable to variation at this upstream step.

## 2. Methods

### Literature Search Strategy

A comprehensive literature search was conducted for this narrative review, covering publications from 1 January 2006 to 31 March 2026. Databases including PubMed and Web of Science were queried using specific keyword combinations. The primary search strings included:

- PubMed: ((“Nitrates”[Mesh] OR “Beta vulgaris”[Mesh] OR “dietary nitrate*”[tiab] OR “inorganic nitrate*”[tiab] OR “beetroot*”[tiab]) AND (“Exercise”[Mesh] OR “Sports”[Mesh] OR “Athletic Performance”[Mesh] OR “exercise*”[tiab] OR “athlet*”[tiab] OR “ergogenic”[tiab] OR “physical performance*”[tiab]));
- Web of Science: TS=((“nitrate*” OR “nitrite*”) AND (“oral*” OR “saliva*” OR “salivary*”) AND (“exercis*” OR “athlet*” OR “sport*” OR “ergogenic*” OR “perform*” OR “endurance*” OR “train*”)).
- Because the primary PubMed string combined nitrate and exercise terms without an oral component, a supplementary PubMed search was additionally run to achieve conceptual equivalence with the Web of Science strategy: ((“Nitrates”[Mesh] OR “dietary nitrate*”[tiab] OR “inorganic nitrate*”[tiab] OR “beetroot*”[tiab]) AND (“oral*”[tiab] OR “saliva*”[tiab] OR “salivary*”[tiab] OR “tongue”[tiab] OR “nitrate-reducing”[tiab] OR “nitrate reductase*”[tiab] OR “microbiom*”[tiab] OR “microbiota”[tiab]) AND (“exercise*”[tiab] OR “athlet*”[tiab] OR “sport*”[tiab] OR “ergogenic*”[tiab] OR “performance*”[tiab])). This supplementary search yielded 231 additional records, of which 17 met the eligibility criteria and were incorporated into the screening process described below. The final search was conducted on 31 March 2026, and the reference lists of included studies and relevant reviews were additionally screened for further records.

**Figure 1:**
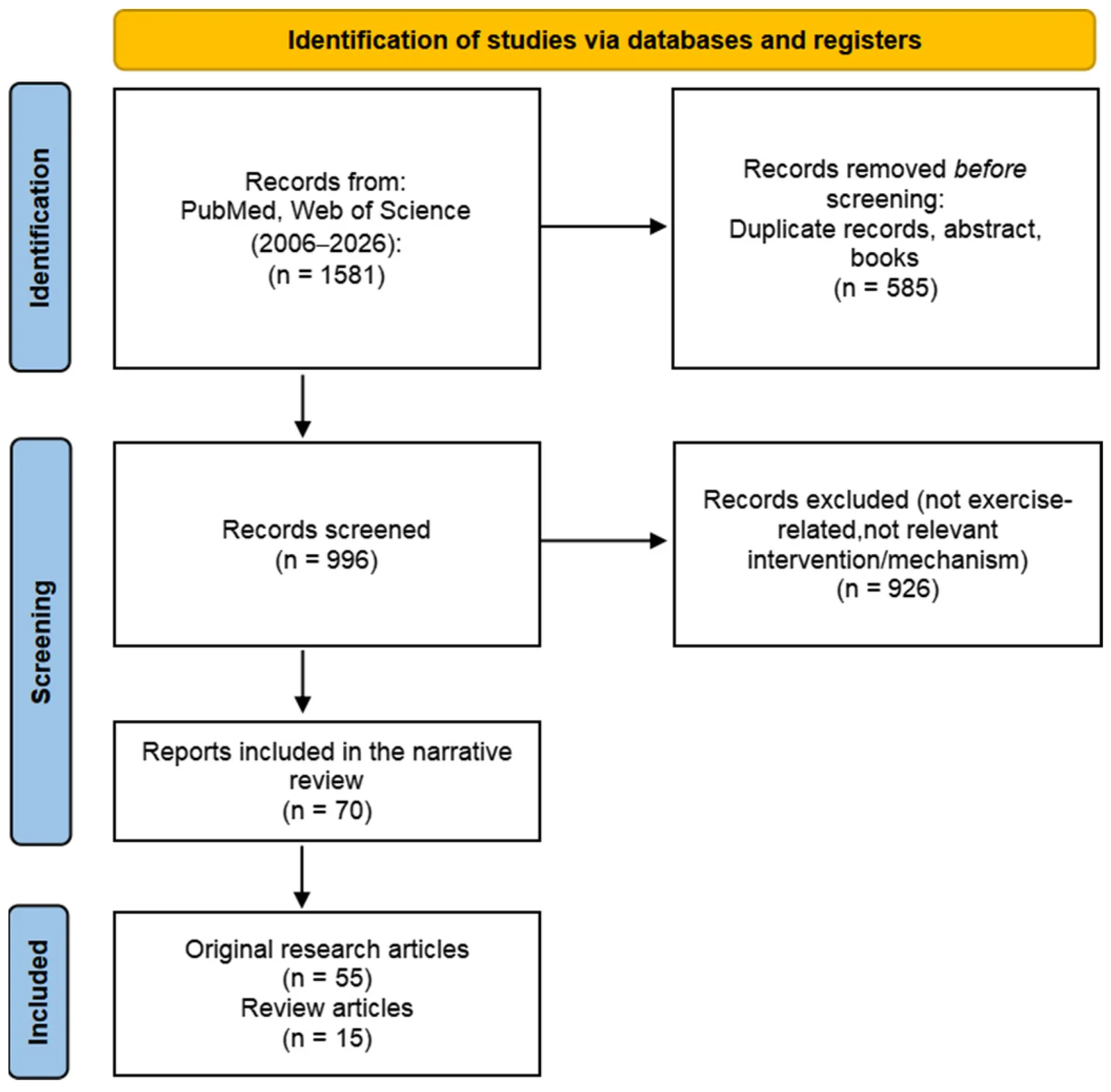
PRISMA diagram illustrating the literature search and selection process.

Our initial search yielded a total of 1581 references. Non-English publications and conference abstracts were excluded. Preclinical in vivo and in vitro studies were eligible as mechanistic support and were interpreted separately from the human evidence within the evidence hierarchy described below. Human supplementation trials, studies investigating antimicrobial mouthwash interference, surveys evaluating oral function, supportive case reports, and preclinical evidence were also included in the review if they were related to nitrate exposure, oral microbial activity, salivary nitrite kinetics, and physiological or exercise performance outcomes. Seventy reports met the eligibility criteria: 55 original research articles and 15 review articles. The original research articles formed the evidence base of the narrative synthesis; human studies directly informing the oral bioactivation–exercise-response hypothesis are summarized in Table 1. Review articles were used for background framing and citation tracking only and were not treated as primary evidence. The search window from 2006 onwards was chosen because the contemporary literature on dietary nitrate and exercise began with the pivotal human trials of Larsen and colleagues in 2007 [5]; this window captures the modern evidence base while keeping screening tractable. Titles and abstracts were screened independently by two reviewers and full texts were then assessed against the eligibility criteria, with disagreements resolved by consensus. Eligible records were peer-reviewed reports published in English; inclusion criteria encompassed randomized controlled trials, observational studies, case reports, and preclinical models (both in vivo and in vitro). Because the central question concerns human response heterogeneity, evidence was interpreted according to an explicit hierarchy: human athlete intervention evidence was given the greatest inferential weight, followed by human non-athlete intervention evidence, observational or clinical mechanistic evidence, and preclinical mechanistic support. The selection process is illustrated using the PRISMA 2020 flow diagram (Figure 1) [47]. This article is a structured narrative review rather than a PRISMA-guided systematic review: we adopted predefined search strings, a PRISMA-style flow diagram, and explicit eligibility criteria, but deliberately did not undertake protocol preregistration (e.g., PROSPERO), formal risk-of-bias grading, or quantitative pooling, because the aim was to test the coherence of a mechanistic pathway across heterogeneous evidence rather than to estimate a pooled effect. A formal meta-analysis was not performed due to substantial heterogeneity in exercise protocols, populations/species, microbiome assessment methods, intervention types/doses/durations, and outcome definitions of fatigue (performance tests vs. biochemical markers), which precluded meaningful quantitative pooling. For each human intervention study, sample size, design features, and the inferential boundary supported by the methodology are summarized in Table 1.

## 3. Oral Nitrate Bioactivation: What Is Established and How It Is Measured

### 3.1. The Enterosalivary Pathway and Its Evidentiary Boundary

Dietary nitrate is not an immediately bioavailable nitric oxide donor. After intestinal absorption, circulating nitrate is actively concentrated into saliva by the salivary glands. Oral commensal microbes then reduce a portion of this salivary nitrate to nitrite, which is subsequently swallowed and absorbed into the systemic nitrite pool. Further reduction of nitrite to bioactive nitric oxide becomes more favourable under relatively hypoxic or acidic tissue conditions. This property makes the pathway particularly relevant to intense and repeated exercise, where tissue hypoxia and acidosis are prevalent [7,30]. The pathway supplements nitric oxide synthase-dependent production; it does not replace it.

Separating exposure from bioactivation is crucial for interpreting study findings. Two individuals receiving the same nitrate dose can convert very different amounts to bioactive nitrite. Mouthwash experiments demonstrate this directly: suppressing oral bacteria blunts the typical rise in salivary and plasma nitrite after a nitrate load [37,38]. This is strong causal evidence for the oral contribution to conversion, but by itself, it says nothing about the magnitude of a subsequent performance benefit.

Several biologically plausible downstream routes could link nitrate bioactivation to exercise effects. Nitrate and nitrite may influence vascular regulation or the oxygen cost of work. Research on skeletal muscle also implicates improved mitochondrial efficiency and altered contractile function [24,25,26]. The relevant mechanism depends on the task: a time trial is not limited in the same way as a maximal sprint, and repeated resistance contractions impose yet another set of physiological demands.

### 3.2. Taxonomic Composition Is Not Nitrate-Reducing Function

Members of several oral genera have been associated with nitrate reduction, most commonly *Neisseria* and *Rothia*, along with *Actinomyces*, *Veillonella*, *Haemophilus*, and *Prevotella*. However, a genus-level taxonomic identification does not establish pathway activity. Species and strains within the same genus can differ substantially in nitrate reductase potential, and enzyme expression also depends on the local ecological context. The functionally relevant question is whether the sampled community actively converts nitrate under the study conditions [33,34,35,36].

Amplicon sequencing describes community composition; it does not measure nitrate reduction activity. A direct functional reduction assay provides a closer approximation of in vivo activity. Paired salivary nitrate and nitrite measurements can add useful contextual information, while metagenomic or metatranscriptomic methods may identify functional genetic potential where feasible. Even so, an association between a specific taxon and salivary nitrite does not validate that organism as a performance biomarker [39,53].

It should also be noted that functional nitrate-reduction assays are not yet supported by fully standardized protocols or published reproducibility data; their results should be read as study-specific functional readouts rather than reference measurements. Throughout this review, ‘oral microbiome’ and ‘oral microbial composition’ refer to taxonomic data, ‘nitrate-reducing function’ and ‘nitrate-reducing capacity’ refer to functional assay results, and salivary or plasma nitrate/nitrite are pathway biomarkers; these measures are related but not interchangeable. Sampling site is another critical methodological consideration. The tongue dorsum is a major nitrate-reducing niche. Saliva, plaque, and buccal swabs sample partly different microbial habitats, and relevant communities are spatially patterned within the mouth. Saliva is practical for monitoring pathway output, but a saliva-only microbial profile should not be assumed to represent the tongue-dorsum community.

### 3.3. What Salivary and Plasma Biomarkers Show, and Where Interpretation Stops

Salivary nitrate confirms that substrate has reached the oral cavity. Salivary nitrite more closely reflects the output of oral bacterial reduction. Plasma measurements sit further downstream and reflect systemic bioavailability. These measures answer different research questions. High salivary nitrate with little corresponding salivary nitrite is compatible with weak upstream conversion. In contrast, a rise in plasma nitrite cannot identify the specific oral niche or microbial activity responsible for its production [39,40,53].

Salivary nitrite is a candidate marker of bioactivation, not a surrogate for an ergogenic effect. Absolute salivary nitrite concentrations and change-from-baseline values are not interchangeable measures of bioactivation, particularly when habitual nitrate exposure differs substantially between individuals [40]. Recent diet and sampling time relative to dosing can shift values appreciably. Saliva flow rate and oral hygiene status also matter, as do hydration status and assay methodology. A robust rise in salivary nitrite makes failed bioactivation unlikely, but it does not establish a change in oxygen cost or exercise performance. Conversely, a null performance result cannot be assigned to failed bioactivation when no pathway biomarkers were collected.

### 3.4. Measurement Timing and Pre-Analytical Control

A salivary measurement is only interpretable when precisely timed relative to nitrate ingestion and exercise. Following nitrate ingestion, plasma nitrate typically peaks within about 1–2 h and salivary and plasma nitrite at about 2–3 h, although both the timing and the magnitude of the peak vary appreciably between individuals [40,49,54]. Nitrate delivery, oral reduction, and swallowing continue dynamically after ingestion, and exercise itself can alter the sampled biochemical milieu. A single arbitrarily timed sample may simply miss the peak response window. Baseline and pre-exercise collections should therefore follow a standardized schedule relative to supplementation. A post-exercise sample can be useful, but its timing must be identical across nitrate and placebo conditions. Tongue-dorsum or saliva samples intended to characterize baseline oral function should be collected before any rinse or disruptive procedure [48,55,56,57].

Pre-analytical control is equally important for valid between-person comparisons. Recent nitrate-rich food intake and habitual diet affect baseline values. Time of day, hydration, and saliva flow alter the sampling context. Oral hygiene practices, tongue cleaning, and antimicrobial exposure can change community composition and activity. Conversely, recent intake of fermented foods or probiotic products may transiently increase oral bacterial load or activity and is likewise worth recording, although direct evidence linking such exposure to altered nitrate-reducing function remains limited. These factors fall into two practical categories: sampling time relative to dosing, pre-test nitrate intake, and restrictions on mouthwash and oral-hygiene procedures should ideally be standardized, whereas habitual diet, hydration, time of day, and saliva flow may be recorded and modelled, because complete standardization of all influences is unrealistic in athlete studies. Crossover trials require sufficient standardization to prevent behavioral variation from being mistaken for a stable oral phenotype. Sample handling and assay choice introduce another source of uncertainty: a low nitrite value may reflect collection or storage conditions rather than weak bacterial capacity [53,55].

A useful sequence of measurements follows from this distinction. Verified nitrate content of the supplement establishes the exposure. Paired saliva samples localize the oral conversion step, while plasma nitrate and nitrite describe downstream systemic availability. A functional reduction assay adds more direct evidence about oral activity. None of these measurements alone identifies a performance responder. Used together, they can locate the first uncertain link in the response chain.

### 3.5. Perturbation Studies Clarify the Upstream Mechanism

Antibacterial mouthwash studies are informative because they directly perturb the proposed upstream step. When oral conversion is suppressed, the usual nitrate-to-nitrite response is attenuated [37,38]. Chlorhexidine also alters overall community composition [58]. The conclusion is mechanistic and quite specific: oral bacteria support efficient enterosalivary nitrate metabolism. Strong antiseptic perturbation is an experimental manipulation, however, and may not reflect the range of naturally occurring variation in oral nitrate-reducing function. Most of these studies were not designed to test athlete performance outcomes.

The applied message should remain narrow. Necessary dental care and prescribed antimicrobial treatment must not be discouraged in an attempt to preserve a hypothetical nitrate response. In research settings, the type and timing of mouthwash use should be fully reported. Recent antibiotic use, oral health status, and tongue cleaning practices may also need to be standardized or recorded [58,59,60,61]. This controls a source of measurement noise; it does not establish microbiome manipulation as a validated performance intervention.

## 4. Does Oral Bioactivation Explain Exercise Responses to Dietary Nitrate?

### 4.1. Direct Evidence Is Currently Sparse

Mouthwash experiments establish the oral contribution to nitrate metabolism, but a harder question remains: do between-person differences at this step explain who benefits during exercise? Most studies cover only part of the pathway. Supplementation trials usually emphasize performance, whereas oral studies tend to stop at nitrate/nitrite kinetics or vascular physiology. The evidence below is therefore presented in descending order of directness: nitrate supplementation combined with oral-function and performance measurement; oral-function perturbation with exercise physiology; oral function related to exercise capacity without supplementation; and non-athlete or clinical response-modifier evidence. This ordering makes explicit how limited the truly direct evidence currently is.

Near-direct mechanistic evidence comes from a 10-day crossover study in older adults. Nitrate-rich beetroot juice altered the salivary microbiome, raised plasma nitrate and nitrite, and reduced the oxygen cost of submaximal walking [35]. These concurrent changes give the result biological coherence, but the design did not test whether the microbial change caused the physiological effect, and it did not involve athletes.

A separate exercise study used mouthwash perturbation and found that lower oral nitrate-reducing activity was accompanied by an attenuated post-exercise blood pressure response and reduced skeletal muscle oxygenation [26]. This links the oral step to exercise-related physiology, but dietary nitrate responsiveness and task performance were outside the study design.

In healthy adults, measured oral nitrate-reducing capacity correlated with peak power output and peak oxygen uptake during graded exercise testing [48]. The study connects a functional oral assay with exercise capacity, but it neither manipulated oral function nor tested the response to nitrate supplementation, so directionality and causality remain open. In trained rowers, variable doses of nitrate-rich beetroot juice likewise illustrate that performance responses to nitrate are heterogeneous and dose- and individual-dependent rather than uniform [62].

The clearest counterexample in athletic populations comes from a small crossover trial in female team-sport players. Acute nitrate-rich beetroot juice produced the expected rise in plasma nitrate and nitrite, but oral community composition did not change, and none of the selected high-intensity performance outcomes improved [49]. This study demonstrates why biochemical exposure cannot be used as a performance surrogate. It did not include a direct assay of oral nitrate reduction. The absence of detectable compositional change after a single acute dose is not unexpected and should not be interpreted as evidence against oral functional involvement, particularly because functional nitrate reduction was not directly measured.

Cross-sectional athlete data add context rather than causality. Competitive athletes differed from inactive controls in tongue-dorsum community composition and had higher salivary and plasma nitrite levels [45]. However, measured oral nitrite production capacity did not differ significantly between groups. Training status may shape the oral background, but the study does not show that this background alters the response to a nitrate intervention.

Repeated nitrate exposure can also alter oral ecology. In older adults, four weeks of beetroot juice changed oral community composition while leaving the intestinal microbiome largely unchanged [50]. The result is evidence of modifiability, not evidence that the community change mediated physiology or improved exercise.

Training itself is another potential influence. Eight weeks of high-intensity interval training altered tongue-dorsum composition and nitrate/nitrite measures in previously sedentary men [63]. No nitrate supplement was given, so the study informs baseline phenotypic differences, not the ergogenic pathway.

Taken across study designs, the missing experiment becomes clear. Mouthwash studies test necessity at the oral step. Functional studies relate oral reduction to exercise capacity. Supplementation trials test later performance outcomes. No athlete study identified in the present search measured oral function directly, collected serial nitrate/nitrite samples, used both a relevant physiological measure and a reliable performance endpoint, and prespecified mediation analysis. The complete pathway has not yet been tested in a single integrated protocol.

A pooled direction-of-effect summary would hide these critical design differences and their inferential limitations. Table 1 instead records what each human study measured and the boundary of inference its design supports. Performance findings count as evidence for oral mediation only when the oral step was assessed in the same participants.

### 4.2. The Performance Literature Supports Plausibility, Not the Oral Causal Claim

The wider performance literature makes an exercise effect plausible, although not universal. Foundational trials reported a lower oxygen cost of work or improved exercise tolerance, and some found improved performance in selected tasks [64,65,66]. Null findings are also common. Evidence syntheses now place the likely effect within a context defined by task, population, and protocol, rather than treating nitrate as uniformly ergogenic [10,13,67].

That literature cannot resolve the oral hypothesis on its own. Many trials omitted tongue-dorsum sampling and did not measure nitrate-reducing activity. Salivary nitrite was often absent as well. A positive performance result is compatible with oral bioactivation driving the effect, but it does not identify the source of the effect. A null result is equally ambiguous: conversion may have been weak, the dose may have been inadequate, or the expected effect may have been smaller than normal test variation.

Generalisability is especially uncertain in trained populations. Endurance and intermittent tests may reveal a benefit when oxygen delivery or recovery between efforts is limiting. A single maximal effort may not respond. Results in elite and female athletes remain mixed, as do findings from high-intensity cycling and resistance exercise [14,15,16,68]. Each setting requires a reliable, event-relevant endpoint.

### 4.3. Alternative and Interacting Sources of Heterogeneity

Oral bioactivation is only one possible modifier of exercise responses. Delivered dose and timing are obvious sources of variation. Habitual diet may change the starting nitrate/nitrite pool, while training status and sex can alter the physiological context in which a response is expressed. In female athletes, menstrual-cycle phase and hormonal contraceptive use may further modulate nitric-oxide-related vascular physiology and should at least be recorded as potential modifiers [69]. Environmental stress, exercise mode, and test reliability add further variation [49]. A strong nitrite response can therefore coexist with a null performance result if the endpoint is insensitive or noisy.

The oral contribution may itself change over time. Repeated nitrate exposure alters oral composition or network features in some studies, but not to the same degree in every cohort [70]. Diet and oral health are plausible influences, as are saliva flow and recent antimicrobial exposure. These factors are better treated as measured effect modifiers than as grounds for assigning a permanent responder label.

Evidence outside sport provides a useful proof of principle. In women with and without hypertension, baseline oral nitrate-reductase activity was associated with the diastolic blood pressure response to a single beetroot juice dose [51]. The finding suggests that oral function can modify a physiological response to nitrate, but transfer to athletic performance remains untested.

Age is another candidate modifier. A crossover study comparing young and older adults found that oral community responses differed by age under nitrate-rich, placebo, and antiseptic mouthwash conditions. Blood pressure fell after nitrate only in the older group, where the change tracked plasma nitrite and a Prevotella-dominated bacterial module [52]. This is informative vascular evidence, not an exercise-performance result.

### 4.4. Evidence-Weighted Conclusion

The evidence is firm at one point: oral bacteria support efficient nitrate-to-nitrite conversion. It is suggestive at the next level, where oral function is associated with nitrite availability and selected physiological measures. The step from oral function to athlete performance remains inferential. Current studies do not show that deliberate microbiome manipulation improves performance, and no oral marker can yet predict an individual benefit.

**Figure 2:**
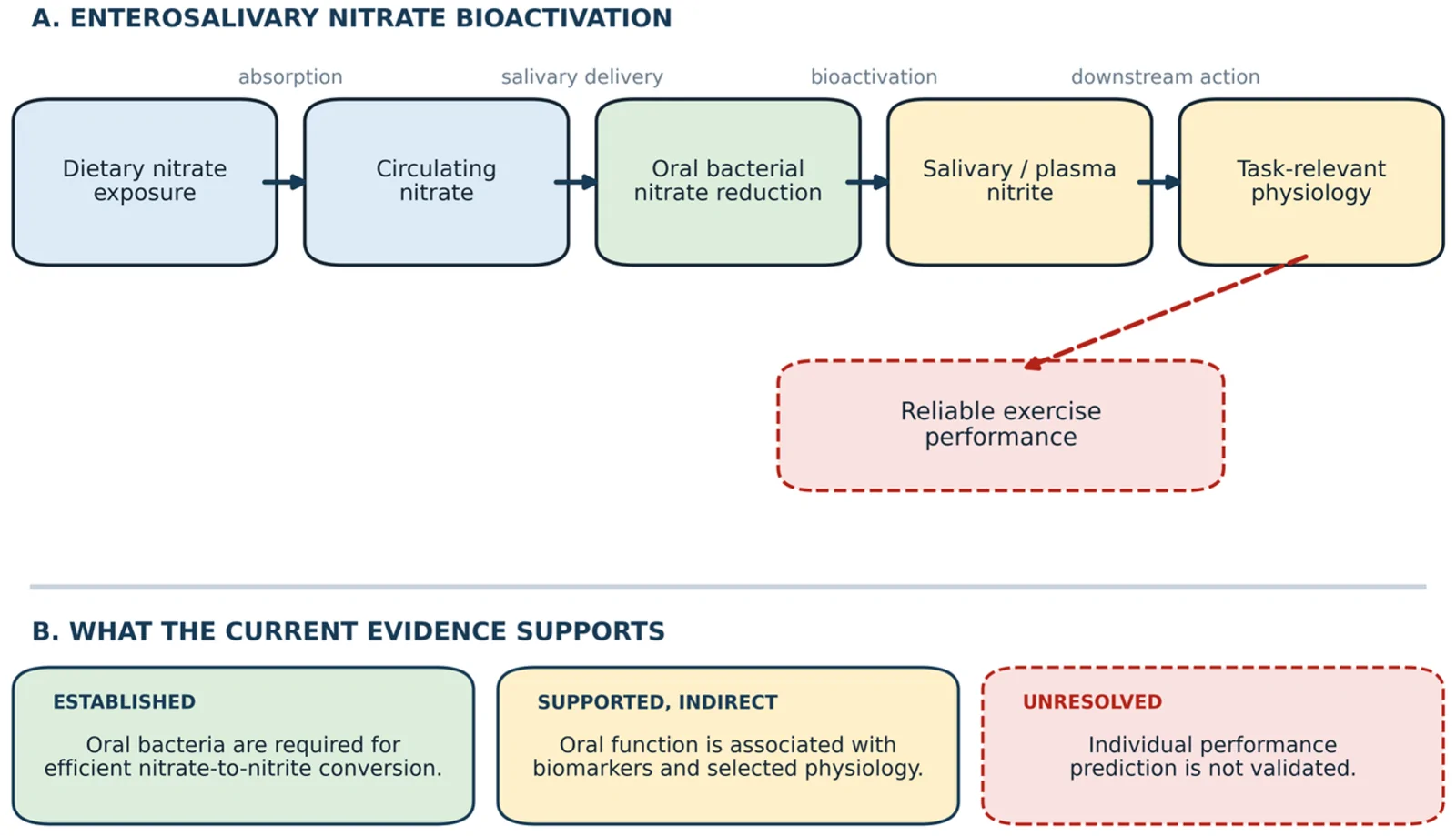
Oral nitrate bioactivation and the evidence boundary for exercise responses. Solid arrows show established biochemical steps in the pathway; the dashed arrow marks the unresolved link between upstream bioactivation and a reliable performance response.

**Figure 3:**
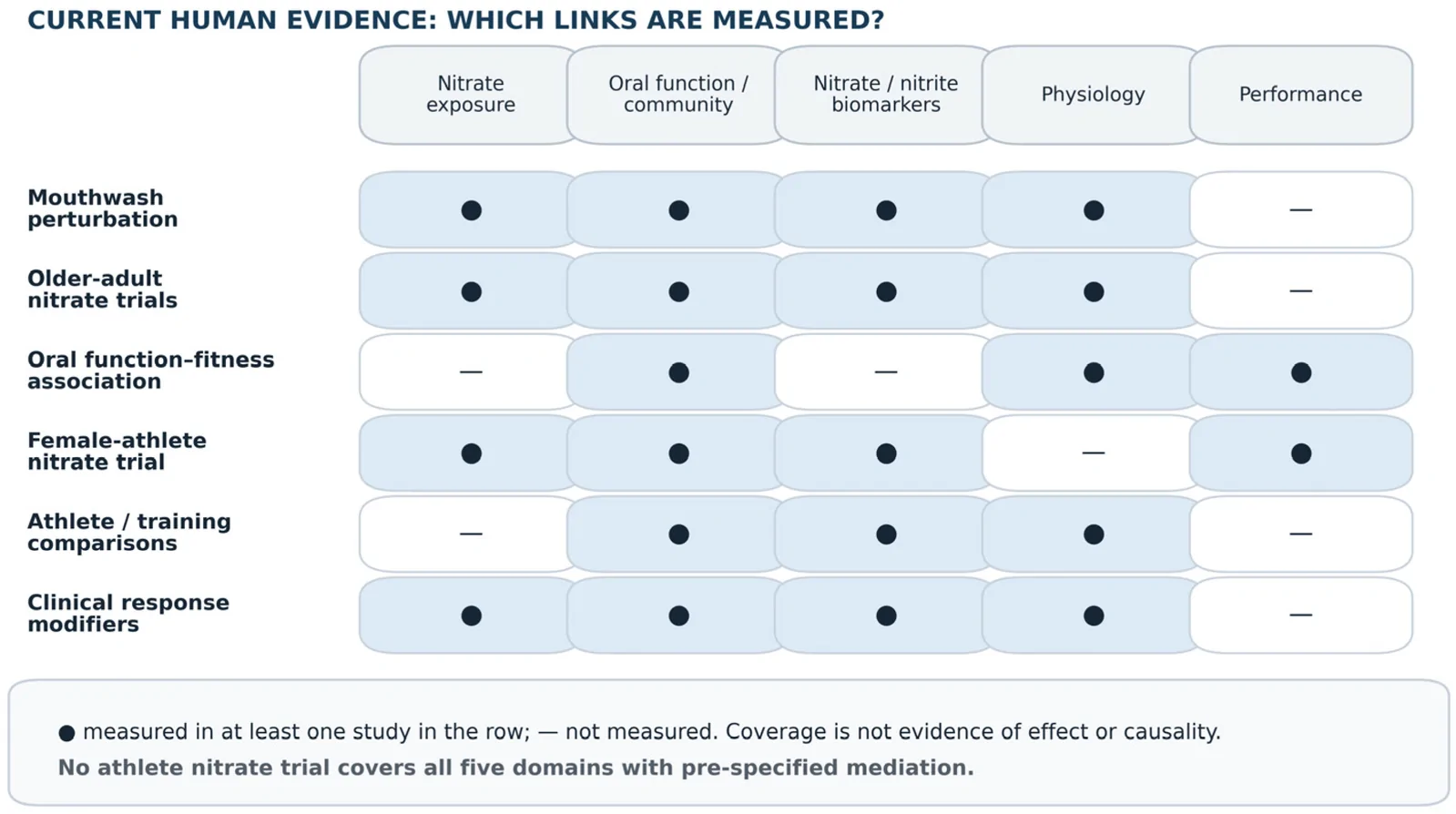
Measurement coverage in the human evidence base. The first rows cover mouthwash perturbation [26,37,38], older-adult nitrate trials [35,52] and an oral function–fitness association [48]. Athlete-specific evidence comes from a female-athlete nitrate trial [49] and athlete or training comparisons [45,63]. The final row represents repeated or clinical response-modifier studies [50,51]. A filled cell means that at least one study represented by that row measured the domain; it does not indicate a positive result or a causal effect. Because each row may represent more than one study, filled cells within a row do not imply that all domains were assessed within the same experiment.

For now, oral nitrate bioactivation is best regarded as a candidate mediator and effect modifier. Figure 2 marks the gap between established biochemistry and performance outcomes. Figure 3 shows how rarely the relevant measurements appear together in human work.

## 5. A Framework for Interpreting Individual Responses

Interindividual variability can mean several different things, and they should not be conflated. First, individuals differ in baseline oral nitrate-reducing function (between-person variability). Second, the same individual does not show an identical biomarker response on every occasion (within-person variability across repeated exposures); critical-difference data indicate that only changes exceeding day-to-day analytical and biological variation can be considered real [40]. Third, physiological responses vary in magnitude even when biomarker engagement is similar. Fourth, performance outcomes add task-specific error, familiarisation, pacing, and motivational variation. Finally, demonstrating true individual response heterogeneity beyond measurement error requires repeated nitrate and placebo exposures, so that a treatment-by-individual interaction can be separated from random within-person variation. The framework below is organised around these distinctions.

### 5.1. Biochemical, Physiological, and Performance Responses Are Distinct

Three levels of response need to be kept analytically separate. A biochemical response is a change in salivary or plasma nitrate/nitrite consistent with pathway engagement. A physiological response sits downstream; examples include reduced oxygen cost or altered muscle oxygenation. A performance response refers to a change in a reliable task outcome, not to either of the earlier levels. A time trial may serve this purpose, as may a familiarised repeated-sprint or muscular-endurance test.

This distinction changes how a result is read. A biomarker rise shows that the upstream pathway was engaged, but it is not a performance finding. A physiological change can still be informative when performance is unchanged. The chosen task may not express that physiology, or the expected benefit may sit within the normal error of the test. Conversely, a performance difference without pathway measurements cannot explain its own mechanism. This framework is an aid to interpretation, not a clinical classification system.

Cardiopulmonary exercise testing can sharpen the physiological level: peak oxygen uptake and gas-exchange indices provide standardised, reproducible information during an incremental test [71,72]. They strengthen phenotyping, but an event-relevant performance test is still needed, and neither index establishes mediation by oral bioactivation.

### 5.2. What Each Profile Can Tell an Investigator

A weak salivary nitrite response after a verified nitrate dose raises a specific possibility: oral bioactivation may have been limited. The result is not diagnostic. Collection time, recent diet, and sample handling must first be checked, as must the analytical method. Repeating the exposure under controlled conditions is more informative than labelling the athlete a non-responder.

A clear biomarker rise with no physiological change shifts attention downstream. The selected physiological measure may have been insensitive, or pathway engagement may simply have failed to alter the relevant tissue response. Both interpretations remain possible until the measurement is repeated with known reliability.

A physiological signal without better performance has a different meaning. The task may not express the altered physiology, and the effect may be too small to matter in practice. This is still a valid biological observation; it is not evidence of an ergogenic benefit.

The most informative test collects all three response levels during repeated, placebo-controlled exposures. Such a design can locate where the chain first weakens. It can also test whether an oral measure predicts the within-person nitrate–placebo contrast. A correlation with baseline fitness cannot answer that question.

### 5.3. Practical Interpretation Without Overclassification

The labels “responder” and “non-responder” often hide substantial measurement uncertainty. A single performance trial is vulnerable to day-to-day noise and familiarisation effects. Pacing strategy and motivation add further variation. Future work should prespecify the main biomarker and performance endpoint, report test reliability, and include repeated measurements where feasible [1,2,3,4,40]. Classifying individuals as responders solely because their change exceeds zero is inappropriate; meaningful-change criteria should incorporate the measurement error of the test, such as its typical error or smallest worthwhile change. Supplementary Table S2 gives cautious interpretations of common response patterns without imposing a fixed cut-off.

Table 1 serves a different purpose. It summarises human studies that directly or indirectly inform the proposed pathway. The emphasis is on what was measured and what the design permits the reader to infer. Broader sport-by-sport summaries are already available in existing nitrate–performance reviews [1,4,22].

### 5.4. What a Credible Individual-Response Claim Requires

A credible individual-response claim goes beyond a difference in group means. The within-person nitrate–placebo contrast must exceed the expected measurement variation of the biomarker or performance test. It should also be reproducible under standardised conditions. Otherwise, regression to the mean or normal pacing variation can be mistaken for a biological effect. Familiarisation is another concern, as is a chance difference in pre-test diet or training. When the claim will guide an athlete’s supplement choices, repeated measurement and an event-relevant outcome become especially important.

Oral measures require the same discipline. Tongue-dorsum composition or salivary nitrite may be useful candidate modifiers. A direct nitrate-reduction assay is closer to functional activity. One baseline value should not be treated as a permanent trait. Distinguishing a true treatment-by-individual interaction from random within-person variation requires repeated nitrate and placebo exposures. A defensible analysis estimates both within-person and between-person variation, then tests whether the oral measure improves prediction of a prespecified physiological or performance contrast [39,40].

## 6. Research Design and Cautious Sports-Nutrition Implications

### 6.1. Minimum Design for Causal Progress

An athlete trial intended to test the pathway should begin with a randomised, placebo-controlled nitrate protocol. A crossover design is usually efficient, provided carryover is addressed with an adequate washout. Dose and timing need to be documented, and habitual nitrate intake should be standardised or recorded. The same applies to relevant oral-care exposure. The oral step must be measured before it can be assigned a mediating role. Sampling should target a defined niche; the tongue dorsum is the most biologically relevant candidate. Salivary and plasma nitrate/nitrite then locate the response further along the pathway. Exercise testing should add one prespecified physiological measure and a familiarised performance endpoint with known reliability. Composition and function should not be collapsed in reporting. Sequencing may identify potential nitrate reducers. A reduction assay or paired biomarker response is needed to test actual activity. Authors should describe the sampling site, collection time, assay method, and recent antimicrobial exposure. Oral health, background diet, and endpoint reliability are also relevant. Supplementary Table S1 provides a concise reporting checklist. The analysis must match the biological claim. Effect modification analysis asks whether a baseline oral measure changes the size of the nitrate–placebo response. Mediation analysis asks a temporal question: did the intervention first alter oral bioactivation, and did that change account for part of the later response? These questions are not interchangeable, and an adequately powered mediation analysis is likely to require a considerably larger sample than typical crossover sports-nutrition studies. Measuring oral composition only after exercise cannot answer this. Nor can a biomarker study without a reliable endpoint. Repeated sampling helps separate pathway signal from analytical variation and informs later sample-size calculations [36,39,40,53,55].

### 6.2. Cautious Sports-Nutrition Implications

Dietary nitrate can be delivered in a standardised beetroot product or through nitrate-rich foods. A manufactured product is easier to dose accurately in an acute trial. Food may fit an athlete’s usual diet better, but its nitrate content is more variable. Acute use and repeated exposure pose different questions; adherence and possible changes in oral ecology become more important over time [22,23,33,34,35,36,54]. Any candidate protocol should be rehearsed in training with a reliable, event-relevant test. Current evidence does not support deliberate oral-microbiome manipulation as routine ergogenic practice [58,59,60]. Avoiding unnecessary strong antibacterial rinse around a research test may remove one source of variation. It is not a reason to stop prescribed treatment or compromise oral health. A probiotic approach would administer live nitrate-reducing organisms; a prebiotic approach would instead favour resident organisms through substrate or dietary exposure. Candidate prebiotic approaches centre on nitrate-rich vegetables (e.g., beetroot, spinach, and rocket) that supply substrate to resident nitrate-reducing taxa such as Rothia and Neisseria [33,34,36]; candidate probiotic approaches, such as nitrate-reducing isolates under investigation for blood-pressure homeostasis [73], remain experimental. Neither strategy has been shown to improve athlete performance [58,59,60,73].

In practical terms, the current evidence base supports four conclusions. (1) Dietary nitrate remains a potentially ergogenic nutritional strategy, with the strongest support in endurance and high-intensity intermittent exercise. (2) Oral nitrate-reducing function is a plausible but unvalidated contributor to response variability; factors such as mouthwash use, recent diet, and sampling timing can influence measured bioactivation. (3) Around research or performance testing, avoiding unnecessary strong antibacterial rinses and standardising pre-test diet and sampling times are reasonable precautions that do not compromise oral health. (4) Deliberate manipulation of the oral microbiome—whether probiotic or prebiotic—cannot currently be recommended as an ergogenic intervention.

### 6.3. Priorities for the Field

**Figure 4:**
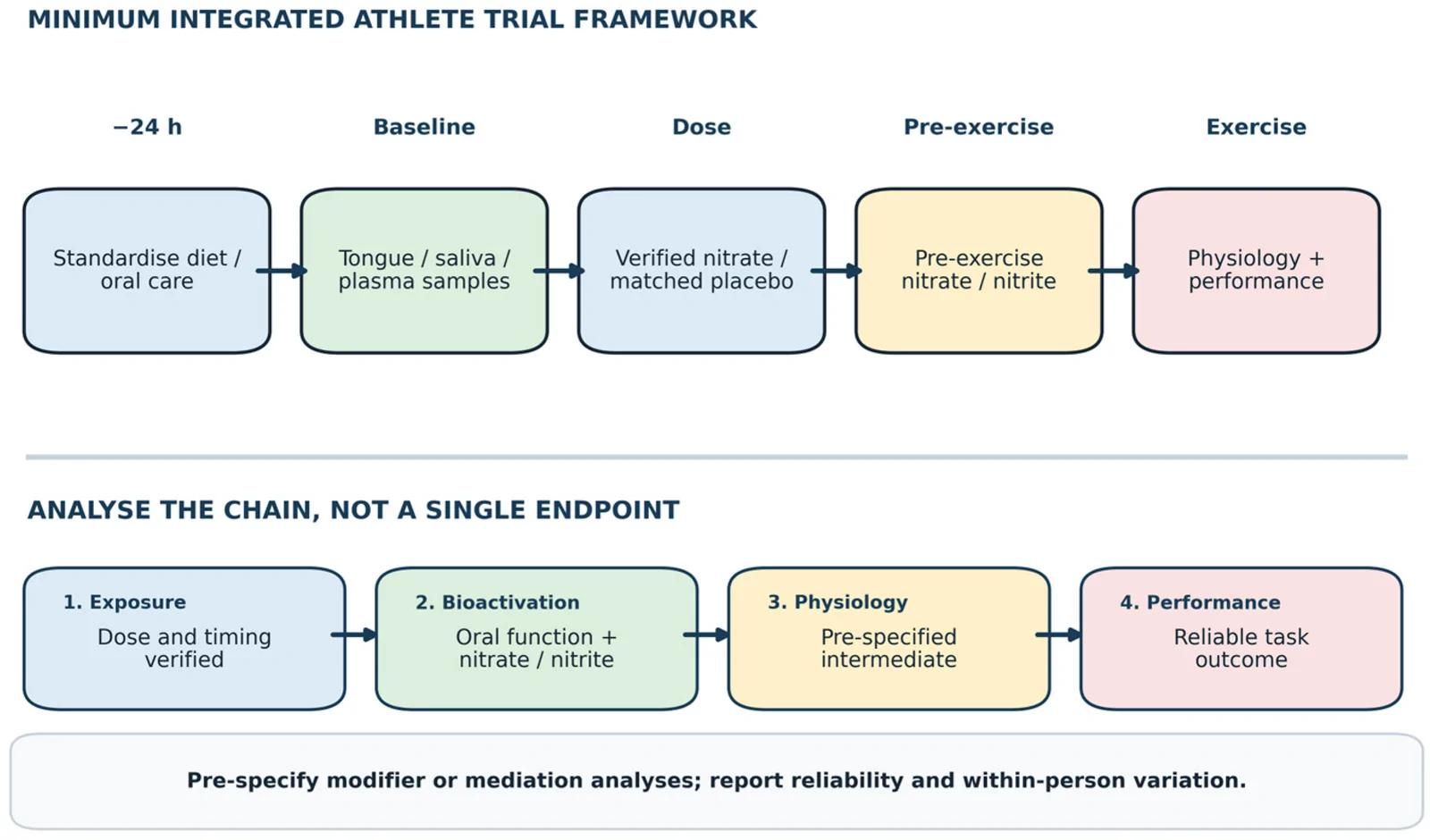
Integrated athlete-trial framework for testing whether oral nitrate bioactivation modifies exercise responses to dietary nitrate. The staged design permits exposure, bioactivation, physiology, and performance to be examined in the same protocol, with effect modification or mediation specified before analysis.

The next studies should recruit populations for whom the exercise test has clear ecological validity. Findings from recreational samples cannot be assumed to generalize to highly trained athletes, and female athletes—who remain underrepresented in exercise and nutrition research—require dedicated investigation [74,75]. Team-sport and resistance-trained groups raise different testing problems and should be studied on their own terms. Because training and repeated nitrate exposure may alter the oral environment [50,63], longitudinal work must distinguish acute supplement effects from adaptation. A second priority is quantifying measurement error. The field does not yet know what size of change in salivary nitrite is meaningful for an individual. The same problem applies to many physiological outcomes. Figure 4 outlines a trial design that can estimate this variation while locating the first weak link in the response chain.

### 6.4. Limitations of the Evidence and Review

Direct athlete evidence is sparse. Much of the mechanistic work comes from healthy non-athletes, and several response-modifier studies involve older or clinical populations. Transfer to performance is uncertain. Studies also differ in oral sampling site and assay used. Nitrate formulation, supplement timing, and exercise tests vary as well. These differences prevent a useful quantitative comparison. Small samples and cross-sectional designs make some taxonomic associations especially fragile. There are also limitations to this review. Its structured narrative design was chosen to test the coherence of a pathway, not to generate exhaustive study counts or a pooled effect. We did not apply formal risk-of-bias or evidence-certainty ratings. Combining heterogeneous evidence types—human athlete interventions, clinical and observational studies, and preclinical work—may also have introduced subjective weighting of mechanistic relative to performance evidence. Prioritising peer-reviewed reports in English may have excluded relevant evidence. The synthesis is therefore hypothesis-generating. It provides neither an oral biomarker threshold nor a validated responder phenotype, and it should not be read as a performance prescription.

## 7. Conclusions

Oral nitrate bioactivation is an essential upstream step in the enterosalivary pathway. Evidence for bacterial conversion of nitrate to nitrite is strong, and biomarker responses vary substantially between individuals. Direct evidence linking oral function to athlete performance remains sparse.

The most defensible position is modest: oral nitrate-reducing function may modify an exercise response, but it does not yet predict one. Athlete trials now need to measure the oral step and follow nitrate/nitrite through to a reliable task outcome. Until such studies are completed, dietary nitrate should be regarded as an ergogenic strategy whose effects vary across individuals and exercise contexts, while the contribution of oral nitrate-reducing function to this variability remains to be established. It is not an established oral-microbiome intervention.

## Figures and Tables

**Table 1:** Human studies that directly or near-directly inform the oral nitrate bioactivation–exercise-response hypothesis. The inference boundary expresses what each design can support; it is not a formal study-quality rating. The table includes all human studies identified in the present search that measured at least two links of the proposed pathway (nitrate exposure; oral microbial composition or function; salivary/plasma nitrate/nitrite; physiological response; exercise performance); ‘near-direct’ denotes studies in which one link—typically the performance outcome or the functional oral assay—was not measured.

Human Study and Population	Design and Oral/Pathway Measures	Physiology or Performance Findings	Inference Boundary for This Review
Govoni et al. (2008), healthy adults (*n* = 7) [37]	Crossover nitrate-load study: sodium nitrate (10 mg/kg) with or without chlorhexidine mouthwash; salivary and plasma nitrate/nitrite measured.	Mouthwash abolished salivary nitrate-to-nitrite conversion and markedly attenuated the rise in plasma nitrite.	Direct causal evidence for the oral step after a nitrate load; no exercise or performance outcome.
Bondonno et al. (2015), treated hypertension (*n* = 15) [38]	Randomised crossover comparison of 3-day antibacterial mouthwash versus water; oral reduction, salivary/plasma nitrate/nitrite, and blood pressure assessed.	Mouthwash reduced oral nitrate reduction and salivary nitrite and increased systolic blood pressure by 2.3 mmHg.	Supports oral-pathway effects on vascular physiology; not a nitrate-supplementation exercise study.
Vanhatalo et al. (2021), older adults [35]	Double-blind crossover (*n* = 30; paired microbiome *n* = 26): 10 days of nitrate-rich beetroot juice (~750 mg nitrate/day) versus depleted placebo; salivary microbiome and plasma nitrate/nitrite assessed.	Oral-community changes co-occurred with higher plasma nitrate/nitrite and lower submaximal walking oxygen cost.	Near-direct biological coherence; neither mediation nor athlete performance was established.
Clarke et al. (2019), healthy adults (*n* = 23; biomarker subset *n* = 15) [26]	Acute moderate-intensity aerobic exercise with antibacterial-mouthwash perturbation; salivary/plasma nitrate/nitrite, blood pressure, and muscle oxygenation assessed.	Mouthwash attenuated post-exercise hypotension and skeletal-muscle oxygenation alongside lower nitrite availability.	Causal support for oral contribution to post-exercise physiology; no dietary-nitrate intervention or performance endpoint.
Thomas et al. (2019), healthy adults (*n* = 50; functional assay *n* = 25) [48]	Cross-sectional salivary assessment and oral nitrate-reduction assay linked with a graded cycling test.	Oral nitrate-reducing capacity correlated with peak power output and peak oxygen uptake.	Useful functional association; no manipulation or nitrate-responsiveness test.
Tan et al. (2024), female team-sport athletes (*n* = 15) [49]	Randomised double-blind crossover: 12.0 mmol nitrate versus 0.1 mmol placebo 2.5 h before testing; oral 16S profile, plasma nitrate/nitrite, sprint, strength, and intermittent-running outcomes assessed.	Plasma nitrate/nitrite increased, whereas oral composition and tested high-intensity performance outcomes did not change.	Key counterexample: biochemical exposure does not itself establish an ergogenic response; direct oral function was not measured.
Simpson et al. (2025), trained athletes versus untrained controls (*n* = 10/group) [45]	Cross-sectional long-read tongue-dorsum/plaque microbiome comparison with salivary/plasma nitrate/nitrite, an oral nitrite-production test, and treadmill peak oxygen uptake.	Athletes differed in oral-community composition and had higher salivary and plasma nitrite; oral nitrite-production capacity did not differ significantly.	Training-status association only; causal direction and nitrate intervention response remain unknown.
Fejes et al. (2025), treated hypertension (*n* = 15) [50]	Randomised double-blind crossover: 4 weeks of 12.9 mmol (~800 mg) nitrate/day versus ≤0.04 mmol placebo, separated by 4-week washout; tongue and intestinal microbiomes assessed.	Oral community composition changed after nitrate exposure, with increased Neisseria and decreased Veillonella; the intestinal microbiome did not change.	Supports oral-community modifiability after repeated exposure; no exercise endpoint and no proof that the community change mediated physiology.
Willmott et al. (2023), women with and without hypertension (*n* = 55) [51]	Single 400 mg nitrate dose in beetroot juice; oral nitrate-reductase activity, tongue 16S profile, salivary/plasma nitrate/nitrite, and blood pressure measured over 2.5 h.	Baseline oral nitrate-reductase activity was associated with baseline diastolic pressure and its response to nitrate.	Response-modifier evidence in a vascular setting; it cannot be transferred directly to athletic performance.
Vanhatalo et al. (2025), young (*n* = 39) and older adults (*n* = 36) [52]	Double-blind crossover with separate 2-week nitrate-rich, nitrate-depleted placebo, and antiseptic-mouthwash conditions; oral microbiome, plasma nitrite, and vascular outcomes assessed.	Oral-community responses differed by age; blood pressure fell after nitrate only in older adults and covaried with plasma nitrite and a bacterial module.	Supports age as a pathway modifier outside sport; mediation and exercise-performance relevance remain untested.

## Data Availability

No new data were created or analyzed in this study. Data sharing is not applicable to this article.

## Supplementary Materials

The following supporting information can be downloaded at: https://www.mdpi.com/article/10.3390/nu18203315/s1, Table S1: Minimum reporting items for exercise studies evaluating oral nitrate bioactivation; Table S2: Evidence-oriented interpretation of common response profiles; no fixed responder threshold is proposed.
