# Supplementary material for: Potential Role of Oral Nitrate Bioactivation in Interindividual Exercise Responses to Dietary Nitrate: A Narrative Review
Source: Nutrients. 2026 Oct 9;18(20):3315. doi: 10.3390/nu18203315 (PMC13708611; doi:10.3390/nu18203315)
Supplement: Supplementary file 1 [file nutrients-18-03315-s001.zip › nutrients-4536563-supplementary.pdf]

Supplementary Material

Oral Nitrate Bioactivation and Interindividual Variability in Exercise Responses to Dietary Nitrate: A Narrative Review

Table S1. Minimum reporting items for exercise studies evaluating oral nitrate bioactivation.

Domain	Minimum reporting item	Why it matters	Recommended interpretation
Intervention	Verified nitrate content, product matrix, dose, timing, duration, and placebo composition	Separates exposure from source/product variability	Report actual nitrate content whenever possible; do not infer exposure from product name alone.
Background diet	Habitual nitrate-rich foods and pre-test dietary standardisation	Background intake can change baseline nitrate/nitrite status	Treat as a potential effect modifier, not a nuisance variable.
Oral context	Sampling site, oral hygiene routine, mouthwash type/timing, tongue cleaning, recent antibiotics, oral health variables	These factors can affect oral nitrate reduction and sample interpretation	Strong antimicrobial exposure should be distinguished from normal oral care.
Oral function	Oral taxonomy plus a functional nitrate-reduction measure where feasible	Taxonomy alone does not establish pathway activity	Pair taxonomic data with salivary nitrate/nitrite or a direct reduction assay.
Biomarkers	Salivary nitrate/nitrite and plasma nitrate/nitrite: collection time, handling, assay method	Locates pathway engagement and downstream availability	Interpret salivary nitrite as a candidate marker, not a performance surrogate.
Physiology	Task-relevant intermediate outcome and its reliability	Tests whether biomarker changes translate into plausible physiology	Select the outcome before data collection; avoid post-hoc mechanism selection.
Performance	Familiarised, reliable, event-relevant endpoint; familiarisation procedures (number and type of familiarisation sessions) and reliability procedures (repeat testing on separate days), with typical error or coefficient of variation reported	Prevents a single noisy test from defining a response phenotype; documented familiarisation and reliability procedures allow readers to judge whether an observed change exceeds measurement error	Report individual and group responses when feasible; judge responder status against the measurement error of the test, not against zero change.
Analysis	Pre-specified mediator/effect-modifier model and handling of repeated measures	Allows a direct test of the oral-bioactivation hypothesis	Do not claim mediation from parallel between-group changes alone.

Table S2. Evidence-oriented interpretation of common response profiles; no fixed responder threshold is proposed.

Observed profile	Most cautious interpretation	What cannot be concluded	Next measurement priority
No salivary/plasma nitrite change after verified nitrate exposure	Possible weak bioactivation, inadequate sampling timing, or assay/standardisation issue	That the participant is a permanent nitrate non-responder	Repeat standardised biomarker assessment; verify oral and dietary conditions.
Biomarker increase without physiological change	The pathway may be engaged without measurable downstream translation in the selected context	That nitrate is physiologically ineffective in all tasks	Use a task-relevant physiology measure with known reliability.
Physiological change without performance change	A biological effect may be present but not expressed in the chosen performance endpoint	That the effect is ergogenic or practically meaningful	Use a familiarised, event-relevant performance test and quantify typical error.
Performance change without oral/pathway measures	A performance signal is observed but the proposed mechanism remains untested	That oral nitrate bioactivation caused the effect	Add oral-function and nitrate/nitrite measures in a replication study.
Variable responses across repeated trials	Responses may be context dependent or within normal analytical/performance variation	That labels based on a single trial are stable biological phenotypes	Model within-person variation and pre-specify meaningful-change criteria.
